# Towards Pathogen-Free Coconut Germplasm Exchange

**DOI:** 10.3390/plants13131809

**Published:** 2024-06-30

**Authors:** Chongxi Yang, Van Anh Nguyen, Naga Prafulla Chandrika Nulu, Sundaravelpandian Kalaipandian, Fernanda Caro Beveridge, Julianne Biddle, Anthony Young, Steve W. Adkins

**Affiliations:** 1 School of Agriculture and Food Sustainability, The University of Queensland, Gatton, QLD 4343, Australia; chongxi.yang@uq.net.au (C.Y.); p.nulu@uq.edu.au (N.P.C.N.); fernanda.carobeveridge@uq.net.au (F.C.B.); julianne.biddle@uq.edu.au (J.B.); anthony.young@uq.edu.au (A.Y.); s.adkins@uq.edu.au (S.W.A.); 2 Queensland Alliance for Agriculture and Food Innovation, The University of Queensland, Brisbane, QLD 4072, Australia; vananh.nguyen@uq.edu.au; 3 Department of Bioengineering, Saveetha Institute of Medical and Technical Sciences (SIMATS), Saveetha School of Engineering, Chennai 602105, India

**Keywords:** coconut, pathogen, phytoplasma, virus, viroid, lethal yellowing, germplasm exchange

## Abstract

Coconut (*Cocos nucifera* L.) is an important palm species that serves as the mainstay of several industries and contributes to the livelihoods of millions of smallholder farmers. International exchange of coconut germplasm has been undertaken for several decades to facilitate the conservation of selected varieties within global genebanks and for the distribution to farmers and scientists. In vitro systems are a convenient and an efficient method for the exchange of coconut germplasm. However, it is possible that these tissue culture systems can transfer lethal pathogens causing a threat to the importing countries. In this review, the following topics are discussed: the major disease-causing agents of concern, the various tissues that could be used for coconut germplasm exchange, and the techniques available for the detection and elimination of disease-causing agents from various transmission systems. Additionally, the lack of clear, science-backed guidelines to facilitate the exchange of in vitro coconut materials is raised, along with recommendations for future studies to ensure the safe movement of coconut germplasm without biosecurity risks.

## 1. Introduction

Coconut (*Cocos nucifera* L.) belongs to the family Arecaceae and is widely grown around the globe in more than 90 tropical and subtropical countries. The palm is versatile, providing numerous useful products for nutrition, medication, and shelter [1,2]. More than 360 commodities can be produced from the palm, and it provides food security and a source of livelihood for a large percentage of the populations of southern Asia and the Pacific region [3]. Recently, the consumption of various coconut products has significantly increased with greater attention given to natural alternatives [4]. The world market for coconut products was estimated to be USD 11.5 billion in 2018 and is expected to reach USD 31.6 billion in 2026, with an annual compound growth rate of 13.6% [5]. However, production is decreasing every year around the world, prominently due to disease and palm senility. Hence, there is an urgent need for mass replanting with genetically superior varieties. This will have the associated benefit of increased production and income for many smallholder farmers who rely on coconut as their main source of income.

Germplasm exchange will continue to play a key role in securing sustainable coconut production [6]. For other plant species, several different plant organs and tissues have been used for germplasm exchange between countries, including seeds, vegetative propagating materials such as tubers, corms, or stem cuttings, as well as various tissue culture-derived materials [7,8]. For the coconut, Samosir and Adkins [9] have described an improved embryo culture technique for germplasm exchange which involved transporting only the embryo of the seed, which is then regenerated into a plant upon receipt in the importing country. In more complex but yet to be adopted methods, Nguyen et al. [10] proposed the movement of cryopreserved tissues or somatic embryogenic cell cultures as vehicles for safe international transfer of coconut germplasm. Such in vitro tissue culture approaches are considered to provide disease-free germplasm for transfer or exchange [11]. Furthermore, in vitro materials have minimal size and weight, facilitating efficient and cost-effective movement, and this is especially important for coconut germplasm exchange [12]. Thus, for the past 30 years, tissue-cultured zygotic embryos have been used as the main approach for efficient international exchange of coconut germplasm [9,13].

Although plant tissue culture systems are expected to isolate tissues free of pathogens or eliminate pathogens during their preparation, recent studies found that the possibility of transmission may occur via an in vitro approach. Such transmission of pathogens through tissue culture systems poses a concern for germplasm exchange and conservation for many species, not just the coconut. To date, only a brief mention has been made in the literature concerning the possible biosecurity issues of coconut germplasm exchange using in vitro systems. Thus, the present review is the first publication on this important subject. The present paper addresses the feasibility of using tissue cultures for safe coconut germplasm exchange with an emphasis on the concerns caused by pathogens. The effective detection and elimination of such pathogens in coconut tissue cultures is also discussed. Further, the review identifies important research gaps that need to be addressed to reduce the biosecurity risks in the international exchange of coconut germplasm.

## 2. The Disease-Causing Agents of Concern

### 2.1. Phytoplasma

Phytoplasmas are obligate intracellular bacterial pathogens that can systemically cause disease in crops and threaten global food safety [14]. Due to their small size (typically ca. 1 µm in diameter), phytoplasma particles can pass from the phloem of an infected tissue to certain kinds of metabolic sink, including those of reproductive organs [15,16,17,18,19]. In the coconut, several phytoplasma types are known to cause devastating diseases, including those of lethal yellowing (LY [20]), LY-like diseases, including Cape St. Paul wilt (CSPW [21]), Bogia coconut syndrome (BCS [22]), Weligama coconut leaf wilt (WCLW [23]) and non-lethal debilitating diseases, including coconut root wilt disease (CRWD [24]). Phytoplasma particles have been detected in different tissues of the coconut palm (Table 1). For example, in infected palms, Gurr [22] discovered BCS in the fruit husk, shell, endosperm, and embryo and in the trunk phloem, while Manimekalai et al. [24] reported CRWD in leaf, inflorescence, and root tissues. A more recent report by Lu et al. [25] have again reported the presence of BCS phytoplasma DNA in the coconut husk, shell, endosperm, and embryos collected from BCS-infected palms. With regards to the vertical transmission of phytoplasma diseases (from seed to offspring), Oropeza et al. [20,26] have reported LY to be present in embryo tissues and in vitro growing seedlings coming from infected palms. Lu et al. [25] have reported the presence of BCS phytoplasma DNA in the leaves and petioles of seedlings derived from BCS-symptomatic coconut and betel nut (*Areca catechu* L.) fruit. However, Manimekalai et al. [24] could not detect CRWD phytoplasma DNA in field-planted seedlings derived from fruit taken from an infected coconut palm.

Many phytoplasma diseases have an influence on both the host plant and their insect vectors, possibly to increase their chances of reproducing and passing on genetic material [27]. One study reported that insect vectors preferred to visit infected plants over non-infected plants, and that certain biotic and abiotic factors (e.g., plant height and air temperature) could influence the number of insect vector visits and therefore aid in the identification of infected plants [28]. Phytoplasma can survive in certain host plants without showing disease symptoms [29]. This disease latency poses an increased likelihood of inadvertent transmission via germplasm exchange. Historically, seed transmissibility of phytoplasma diseases in plant species was thought to be impossible; firstly due to the early abscission of the fruit or seed before infection could take place, and secondly because of the poor phloem connection that exists between the forming zygotic tissues and the mother plant [30]. However, it has since been shown that vertical transmission of some diseases can occur in many crops [31,32,33]. For example, vertical transmission of phytoplasma occurs in apricot (*Prunus armeniaca* L. [34]), maize (*Zea mays* L. [35]), canola (*Brassica napus* L. [36]), and tomato (*Solanum lycopersicum* L. [37]).

**Table 1:** Major coconut diseases, their causal agents, known localization within the palm, and symptoms.

Agent	Disease	Localization	Symptoms	References
Phytoplasma	Coconut lethal yellowing disease (LY)	Husk, shell, endosperm embryo, plumule, phloem cells and haustorial tissue from diseased coconut palms	Untimely shedding of fruit, discoloration in younger leaves, and darkening of the inflorescence, finally leading to the death of the coconut palm.	[22,38,39]
	Coconut root wilt disease (CRWD)	Leaves, inflorescence, and roots	Flaccid bending of leaflets, accompanied by leaf yellowing, necrosis, compromised stomatal regulation, and damage to the root system.	[24]
	Weligama coconut leaf wilt disease (WCLW)	Sieve tubes, leaflets	The leaves exhibit flaccidity and marginal necrosis, accompanied by intense yellowing of the fronds. With disease advancement, the crown diminishes in size, and the trunk starts to taper. In addition, the female flower production declines, as does the palm’s productivity. Moderately affected palms show necrosis in the root tips, while severely affected palms lack young roots. Some of the infected palms also exhibit leaf rot disease. However, no studies have reported the death of a coconut palm caused by this disease.	[23,40,41,42]
	Cape St. Paul wilt disease (CSPW)	Fronds, inflorescence, and leaves	Premature nut drops, yellowing of fronds, and necrosis of immature inflorescences, followed by progressive yellowing of the crown from the older leaves upwards, and death of palms.	[43]
	Bogia coconut syndrome (BCS)	Fruit husk, shell, endosperm, embryo, trunk phloem	Premature dropping of fruit of all ages. The outer fronds of the crown will droop and develop a pale yellow colour. Fronds then turn brown and hang down the stem, like a skirt. A dry rot develops in the newly expanding spear, progressing downwards to the growing point. Complete necrosis—death of the palm.	[22]
Virus	Coconut foliar decay virus disease (CFDV)	Leaflet, frond, phloem, tissue adjacent to and within necrotic zones	Yellowing in certain leaflets below the unfolding spear leaf. Then, a broader yellowing occurs in both the affected fronds and neighboring organs. Synchronously, these fronds undergo a lateral necrosis near the petiole’s base, resulting in their collapse.	[44]
Viroid	Coconut cadang-cadang viroid disease (CCCVd)	Vascular tissues, nucleolus of mesophyll cells, embryos.	The fruit assumes a rounded shape, displaying distinctive scarifications at its equator, while the initial non-necrotic, translucent, bright yellow leaf spots emerge. Inflorescences undergo necrosis, halting fruit production, slowing down new frond development, and causing larger and more frequent leaf spots. Later, fronds start to appear chlorotic when observed from a distance. Finally, preceding death, leaf spots merge, and the entire crown exhibits a distinct yellow or bronze hue, significantly reduced in size with a diminished number of fronds.	[45,46,47]
	Coconut tinangaja viroid disease (CTiVd)	Unreported	A decrease in frond quantity, along with diminished size and quantity of fruit, leads to the shriveling and deformation of the fruit. This results in the cessation of fruit production, failure to generate inflorescences, tapering of the distal end of the trunk, persistent stipules, stippling of leaflets characterized by fine chlorotic spots, and the development of brittleness in both leaflets and fronds.	[48]

In summary, phytoplasma diseases are the most lethal and destructive plant diseases due to their cryptic and unique mode of transmission and their impact upon productivity. Since recent studies have shown some phytoplasma to be seed-transmitted in several plant species, this raises the concern for coconut germplasm exchange through an embryo culture.

### 2.2. Viruses

Viruses consist of a small DNA or RNA genome surrounded by a protein coat [49]. Coconut foliar decay virus (CFDV; Table 1) is a small circular single-stranded DNA virus and was the first virus to be characterized in coconut palms [50]. It causes lethal coconut foliar decay disease (CFD) and is usually transmitted by the leaf-feeding planthopper, *Myndus taffini* [51]. The CFDV DNA has been detected in the husk of germinating fruit, but not in the resulting seedling leaves [44,50]. In another study, the DNA of CFDV has been detected in zygotic embryos obtained from a diseased coconut palm [50]. However, further transmission from the embryo to the seedling has not been observed [44,50]. According to Pagán [52], the presence of viruses in seeds does not mean they will be transmitted to the progeny plants because many viruses are found only in the testa or endosperm tissues and are not present within the embryonic tissues that participate in the formation of the seedling. However, the existence of a virus in a seed or a fruit still poses a concern for transmission to progeny. Johansen et al. [32] suggested that when there is unsuccessful transmission of viruses through the seed, it may be because the virus is inactivated during the development of the embryonic tissues under physiologically active conditions. 

In summary, the evidence suggests that the transfer of lethal coconut viruses by intact fruit may be possible, but there is no evidence of virus transfer through tissue-cultured coconut embryos. It should be noted that the possibility of viral detection could be enhanced by using more modern and sensitive techniques, which are described in the following Section 4. Thus, until determined otherwise, embryo cultures are an effective way of exchanging coconut germplasm without the introduction of viral diseases.

### 2.3. Viroids

Viroids are small single-stranded, circular RNAs that can act as infectious pathogens [53] and, unlike viruses, have no coat protein and are confined to infecting angiosperms. Viroids have been reported to be transmitted through the seed and pollen, through mechanical means (e.g., on contaminated farming tools), or by co-transmission with fungal diseases [54,55]. Viroids, such as the Coconut cadang-cadang viroid (CCCVd) from the Philippines [45] and the Coconut tinangaja viroid (CTiVd) from Guam, have been reported to be responsible for lethal diseases in coconut palms ([48], Table 1). Transmission of viroid diseases through the seed and pollen have been observed in plants such as chrysanthemum (*Chrysanthemum indicum* L. [56] and eggplant (*Solanum melongena* L. [57]). In the coconut, palms infected with CCCVd have been reported to transmit the disease through pollen, and CCCVd can be detected in both embryos and in vitro germinated seedlings [46]. In addition, CCCVd variants have been shown to be transmitted from infected palms to in vitro plantlets in oil palm [58]. In the case of propagation through tissue cultures, oil palm (*Elaeis guineensis* Jacq.) ramets have been shown to be a potential way of spreading systemic viroid diseases [59].

In summary, studies have indicated that certain viroids can be transmitted via pollen and seed and have been detected in coconut embryos and in vitro germinated seedlings coming from those embryos. Thus, it is important to test for the presence of viroids in embryos if they are used for germplasm exchange.

## 3. Tissues Used for Coconut Germplasm Exchange

### 3.1. Zygotic Embryos

**Figure 1:**
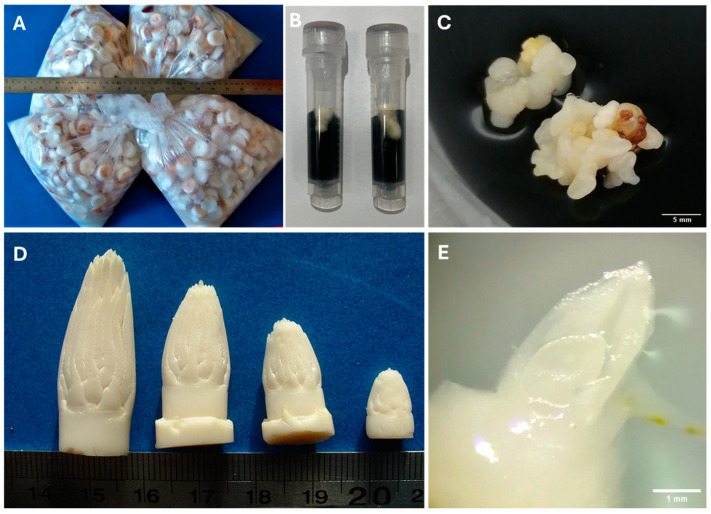
Coconut tissues being or having potential to be used for germplasm exchange. (**A**) Coconut endosperm plugs containing embryos being transported in poly bags. (**B**) Isolated coconut embryos being transported in vials (1.5 mL). (**C**) Coconut embryogenic callus on an induction medium. (**D**) Coconut inflorescences isolated at different stages of development (**E**) Coconut apical meristem. Images were kindly provided by Sisunandar Sudarma (**A**,**D**), Chongxi Yang (**B**), Eveline Kong (**C**), and Steve Adkins (**E**).

Zygotic embryo cultures have been successfully developed as a primary way for the exchange of coconut germplasm (Figure 1). This technique has also been used for many other vital purposes such as coconut germplasm collection and conservation [10,60,61]. There have been some reports of in vitro cultured embryos carrying lethal pathogens acquired from infected germplasm [20,22,45]. However, no studies have yet displayed full vertical transmission of those pathogens to progeny plants. The most comprehensive studies on the coconut so far have shown LY phytoplasma DNA to be present in embryos [20] and in plumule and haustoria tissues [38], while CCCVd has been detected in both embryos and in vitro germinated seedlings [46]. These studies indicate that further work needs to be undertaken to ensure that the method of using coconut embryo cultures is a safe method for germplasm exchange.

### 3.2. Apical Meristems

Coconut apical meristem cultures have not been used as a method for pathogen-free germplasm exchange to date, although they have great potential as a method to transfer germplasm, as meristems are often pathogen-free (Figure 1). Meristem cultures and transfer have been shown to be an effective method for, firstly, the elimination of latent plant pathogens from diseased tissues and, secondly, as a suitable method for germplasm exchange for many other species, including cassava (*Manihot esculenta* Crantz. [62]), sugarcane (*Saccharum* spp. [63]), and potato (*Solanum tuberosum* L. [64]). The actively dividing cells of the meristem, with no direct vascular or plasmodesmata connections to the main body of the plant, enable the meristem tissues to remain pathogen-free [65]. However, care must be taken to avoid dissection of differentiated tissue within which pathogens may be present. Meristems also contain naturally high levels of auxins that are thought to decrease viral activity [66,67,68,69]. Another advantage is that the meristem culture is a more genetically stable tissue than the callus or somatic embryogenic callus cultures, thus giving better chances of genetic fidelity being maintained in the transferred germplasm. Apical meristems, isolated from in vitro coconut seedlings, have been successfully regenerated into soil-growing coconut plants (Figure 1) [70]. Thus, it is now possible to consider the international exchange of coconut germplasm by using meristem cultures.

In summary, although the apical meristem culture has not been used for pathogen elimination in the coconut, many studies have reported the successful application of meristem cultures for pathogen elimination in other crops. The apical meristem culture would be an effective method for pathogen elimination in the coconut; however, it requires specialized skill to isolate and recover the seedlings when compared to the embryo culture and recovery method.

### 3.3. Somatic Embryos/Embryogenic Callus

In some instances, it has been reported that certain pathogens can be transmitted through callus cultures (Figure 1) and plantlets produced by somatic embryogenesis (SE) procedures [71,72]. In contrast, there have been several reports showing SE to produce plantlets free of pathogens; for example, Parmessur et al. [73] and Gambino et al. [74] reported on the sugarcane and grapevine (*Vitis vinifera* L.), respectively. This has been explained by vascular tissue in the developing somatic embryos, not being connected to the original explant tissues and therefore pathogen transfer not taking place.

Several studies have reported different degrees of eradication of disease-causing agents from infected explants by SE [75,76]. In the coconut, the main explant used to produce SE plantlets is the plumule [77], but this has been shown to carry LY phytoplasma DNA [20] if isolated from infected palms. In addition, a method for the selection of pathogen-free callus or somatic embryos has not been developed, so exchange by these two approaches is not suggested until further work is undertaken on the detection of pathogen-free cultures.

In summary, pathogen transmission through SE varies from species to species, but it has not been studied in the coconut. Transmission depends on tissue size and infection status. While some studies report pathogen-free SE plantlets, others found mixed results, emphasizing the need for further research before this method could be considered for coconut germplasm exchange.

## 4. Methods for the Detection of Lethal Pathogens

### 4.1. Detection Using Artificial Transmission Test Systems

Methods for testing pathogen transmissibility using whole plants will always pose challenges for large, relatively long-lived plants such as palms. Virus and viroid detection methods using highly susceptible and symptomatic test plants have been used for sweet potato (*Ipomea* spp.) but are lacking for many palm species. Several systems have been developed for phytoplasma detection using either whole plants or in vitro tissues from a range of other plant species [78,79,80]. In vitro transmission tests are a more rapid alternative to the cumbersome testing of field-grown plants. The in vitro approach is more cost- and labor-effective and can better facilitate the use of controls. Transmission tests have been attempted on field-growing coconut for a planthopper-transmitted phytoplasma disease but have not been successful [81]. However, improved phytoplasma transmission tests have been described for the testing of healthy palms (e.g., Manila palm; *Veitchia merrillii* (Becc.) H.E.Moore) when caged with the infecting insects [82] or by caging infected and healthy young coconut palms together with the suspected vectors [43]. Other phytoplasma transmission tests have been developed for in vitro growing coconut seedlings when enclosed in glass vessels with the suspected phytoplasma-transmitting vector [20,83]. Interestingly, one study on in vitro cultures of the pear (*Pyrus* spp.) has shown that once infected, the cultures can carry the phytoplasma particles for years [84]. The same study also reported that the concentration of phytoplasma particles in in vitro culture can exceed that of field-grown plants. 

In summary, no phytoplasma transmission tests so far have shown coconut tissues to transmit any form of phytoplasma diseases to the next generation of palms.

### 4.2. Visual Detection Methods

Since revealing the Tobacco mosaic virus (TMV) in 1939, the transmission electron microscope (TEM) has been the tool of trade for traditional virology [85]. The TEM has been used effectively to observe and localize the presence of certain coconut viroids and viruses (e.g., CCCVd [47] and Areca palm necrotic ringspot disease virus (ANRSV) [86]). The first visual detection methods for phytoplasma were those involving the use of the fluorescent stain 4′,6-diamidino-2-phenylindole (DAPI). This was the first method to be found to stain DNA which could then be observed under ultraviolet light for adenine-thymine-rich regions [87]. Early DAPI staining studies were able to show that most phytoplasma particles were localized in the sieve tube elements of the phloem in infected coconut plants [88].

More recent remote sensing methods, applied to in situ field-growing populations of plants, can collect within- and between-field epidemiological data based on expressed symptoms without killing the plant materials to be tested. Such methods involve image processing and machine intelligence [89,90,91], which use unmanned vehicles to collect remote radiated images from the plant population, which are then processed under machine learning algorithms, enabling researchers to timely detect the presence of symptoms that would be impossible to detect with the naked eye. However, these remote sensing methods must be thoroughly aligned with the presence of the pathogen, which should not be confused with other factors such as plant stress, nematode activity, or nutritional deficiencies.

### 4.3. Immunological Detection Methods

Immunological detection methods are based on the binding of polyclonal antibodies to a pathogen that can result in its visual detection. This approach is suitable for the detection of a wide range of plant pathogens, from bacteria to viruses [92], but are not considered to be as sensitive as molecular methods that target specific nucleic acid sequences. The direct antigen coated-indirect enzyme linked immunosorbent assay (ELISA) has been used to detect CRWD phytoplasma present within coconut tissues rapidly with high sensitivity and specificity (96–98%) [93]. Further optimization has been undertaken to ensure that the method can now be routinely used to detect CRWD outbreaks and aid in their management [94]. In another instance, an ELISA assay has been used for the detection of WCLD phytoplasma, with reported accuracy above 90% [95]. Differential tissue responses to antibiotic treatments have also been used as a method to detect phytoplasma from non-phytoplasma bacterial diseases [96,97].

### 4.4. Molecular Detection Methods

To date, several molecular diagnostic methods have been developed for the detection of pathogens, including phytoplasma, viruses, and viroids. In recent decades, considerable progress has been made with nucleic acid-based technologies worldwide, with an exponential increase in the number of protocols with enhanced specificity, sensitivity, and reliability to detect plant pathogens present even below potential thresholds [98]. In the process of increasing the feasibility for routine use, focus has also been placed on time and cost reductions. Additionally, methods that enable high-reproducibility and high-throughput screening without the use of toxic and radioactive chemicals are most preferred [99]. The availability of nucleic acid sequence information provided access to several nucleic acid-based diagnostic methods. The molecular diagnostic methods used for pathogen detection especially in the coconut are discussed further.

#### 4.4.1. Molecular Hybridization Assays

Robust and reliable molecular hybridization assays have been developed using nucleic acid complementarity to known targets. These include Southern blots that rely on DNA and Northern blots that use RNA and are widely used for pathogen detection in diseased plants. For example, the presence of phytoplasma nucleic acids has been detected by dot-blot hybridization approaches in both symptomatic and asymptomatic plants and in in vitro cultures [80]. The CCCVd has been detected using two-dimensional polyacrylamide gel electrophoresis (2D-PAGE) and Northern blots as a weak signal, indicating the presence of a low concentration in infected oil palm plants [100]. Blotting methods are generally very specific but time-consuming and require a high level of stringency for optimal results.

#### 4.4.2. Polymerase Chain Reaction (PCR)-Based Methods

These methods comprise PCR along with its variants, including the reverse transcriptase polymerase chain reaction (RT-PCR), nested PCR, and real-time qPCR and are being increasingly employed to detect plant pathogens accurately and quickly at low concentrations.

(a) Conventional PCR: The molecular detection of phytoplasmas present in symptomatic tissue is routinely undertaken by the PCR using phytoplasma-specific universal or phytoplasma group-specific primers designed based on the highly conserved 16S ribosomal RNA (rRNA) gene sequences, the ribosomal protein, and elongation factor genes [101]. Phytoplasmas can be easily detected using PCR with the highest sensitivity in immature rather than mature tissues [101], as evidenced by the detection of LY phytoplasma in coconut leaves [102] and the Coconut yellow decline (CYD) phytoplasma in spear leaves, inflorescences, or trunk tissues of affected symptomatic and asymptomatic coconut palm varieties [103]. A high population of viroid-like molecules has been identified using PCR coconut palms infected with the Coconut tapering disease in Sri Lanka [104]. Similarly, a sense-specific single-primer PCR assay has been employed to identify CFDV DNA from coconut palms in Vanuatu [105].
(b) Reverse transcriptase-PCR (RT-PCR): As most plant viruses have RNA genomes (and even DNA viruses produce RNA transcripts), it is most effective to detect virus infections by analyzing RNA sequences from infected plant samples [106]. The RT-PCR technique has been employed for detecting low concentrations of viruses and viroids in infected plants, with the only requirement being that of obtaining good quality RNA [106]. The application of the RT-PCR technique has successfully helped to detect the CCCVd viroid variant in oil palm leaf tissues [99], the Areca palm necrotic ringspot virus (ANRSV) in areca palm (*Areca catechu* Linn. [86]), and African oil palm ringspot virus (AOPRV) in infected oil palm leaves [107].
(c) Nested PCR: Nested PCR methods, where four primers are used in two consecutive rounds of DNA amplification, have greatly enhanced specificity and sensitivity, and therefore aid in the detection of a low titer of pathogens [108,109]. Most of the nested PCR protocols developed have been focusing on phytoplasma detection and are used to increase the sensitivity of the available protocol [98]. Nesting of universal group primers with group-specific primers has helped in the diagnosis of phytoplasma infection in well-defined taxonomic groups [110]. For example, the WCLW-causing phytoplasma has been successfully detected using an optimized nested PCR in symptomatic coconut plants in Sri Lanka [111]. Similarly, CRWD- and CSPW-causing phytoplasma have also been detected in coconut palms using nested PCR [15,24,112].
(d) Quantitative PCR (qPCR): In recent years, qPCR has proven to be an indispensable tool for the molecular diagnosis of pathogens. It is about 10 times more sensitive than standard PCR and does not require gel electrophoresis for target confirmation. The qPCR can simultaneously detect and quantify the pathogen, and this method is less affected by cross-contamination and is less work-intensive as compared to RT-PCR and nested PCR. For example, phytoplasma belonging to the 16SrIVgroup causing CRWD have been easily and effectively detected by qPCR [113], especially when compared to conventional and nested PCR [114]. The qPCR approach using the TaqMan probe gives higher sensitivity than nested-PCR when detecting the 16SrIV subgroups of the LY phytoplasma in the coconut [83,115,116]. The CCCVd variants in oil palm have been detected by using the qPCR technique [117]. This technique could be used in the coconut as well.
(e) Digital PCR: Digital PCR (dPCR) is a breakthrough next-generation PCR technology, and it works by partitioning the sample into thousands of separate reaction compartments, conducting single, parallel PCR reactions [118]. It is used to detect phytoplasma and viruses, with several advantages over qPCR diagnostic assays, including higher sensitivity, precision, accuracy in detecting extremely rare target sequences, absolute quantification without standard curve and reference samples, greater tolerance to PCR inhibitors, and suitability for the preparation of in-house reference materials [119]. Reverse transcription (RT) dPCR has been used to detect viruses and viroids in apple and citrus plants [120,121], and this technique can be used for the coconut.

#### 4.4.3. Isothermal Amplification Techniques

Isothermal amplification techniques (IAT) are considered to offer the advantages of being cheaper, faster, reliable, more robust, and adequately sensitive with minimal sample processing when compared to PCR-based approaches [122,123]. In addition, another advantage is that they can be conducted in the field. The IATs produce high copy number of DNA products by using strand displacement activity to achieve DNA amplification and can be performed in a thermostat water/dry bath at a constant temperature, without the need of a thermocycler [124,125]. Loop-mediated isothermal amplification (LAMP), recombinase polymerase amplification (RPA), and rolling circle amplification (RCA) are several IATs that are available as kits, making pathogen detection easier [123]. The RPA approach enabled the detection of the CCCVd sequence in infected coconut and African oil palms [126,127]. The RCA, with a deep sequencing method, helped in the identification of CFDV DNAs sequences from symptomatic coconut leaves [128]. The approaches of 16S rDNA-targeted LAMP and real-time LAMP have been undertaken to detect CRWD and CFDV [128,129]. The approaches have been found to be more robust when compared to nested PCR, implying the potential of these methods in the rapid screening and detection of phytoplasma in large field samples [129]. From the available studies, it has been suggested that RT-LAMP can be used for routine virus or viroid detection from field samples. 

#### 4.4.4. Next-Generation Sequencing

Currently, next-generation sequencing (NGS), or deep sequencing technologies, enables unbiased and hypothesis-free testing of different plant samples and is being widely used for simultaneous detection of multiple viruses [106,130]. Combining NGS with a tissue culture for plant virus detection has been conducted successfully in yam (*Dioscorea* sp.) for isolates of badnavirus and potyvirus [131]. This is an extremely sensitive technology allowing for the generation of massive amounts of sequence data. The NGS has incredible multiplexing potential to simultaneously screen and unbiasedly detect the genome sequences of multiple RNA and DNA viruses, viroids, and phytoplasma present in host plants, which could not be detected using previously developed biological, serological, and molecular tools.

### 4.5. Limitations of Current Detection Methods

The development of tools for the detection of lethal pathogens has proven to be difficult, with each test having its strengths but also some weaknesses. For example, visual detection methods cannot distinguish between viable and non-viable phytoplasma particles [38], complicating the interpretation when using this detection approach. The main limiting factor of using ELISA assays, when applying them as immunological assays, is their cross-reactivity with non-specific antigens, leading to false positive results, and the time for generating results, which is unsuitable for field screening purposes. So far, molecular diagnostic methods have proven to be much more sensitive, specific, faster, and reliable methods for pathogen detection when compared with other detection methods. However, these tests also have their own share of limitations. Molecular hybridization assays have the advantage of not giving false positives and cross-contamination risks; however, these methods are laborious, time consuming, and require large quantities of tissue samples. 

Conventional PCR, along with its variants such as RT-PCR and nested PCR, can also give false positives due to cross-contaminations with the phytoplasma and other bacterial genomes [132]. In addition, post-PCR requirements of gel electrophoresis of the PCR products require visualization, and this can be time-consuming [114], especially when dealing with large sample numbers. Although LAMP assays are highly suitable and economical for field detection of larger numbers of samples in a short period of time, they too have the disadvantages of giving false positives, having complex steps in primer designing. Due to these two reasons, multiplexing LAMP assays have not been developed [132]. On the other hand, in one recent study, NGS did not detect viruses or viroids in low concentrations, which can be easily detected by RT-PCR [133]. Thus, it is recommended to use at least two different approaches for pathogen detection [133] if possible. The use of molecular detection methods is further limited, especially in developing countries, by the lack of skilled personnel and appropriate facilities to conduct such tests. 

## 5. Methods for the Elimination of Lethal Pathogens

### 5.1. Chemotherapy

Antibiotic treatments are among a wide range of approaches under the umbrella term of “chemotherapy” (usage of chemical substances to destroy or inhibit the growth of pathogens in plant tissues). The use of antibiotics in agriculture to manage and eliminate lethal bacteria is well known [134,135]. However, less is known about their effects on phytoplasma diseases. In the coconut, the management of the LY phytoplasma disease has been achieved with the use of oxytetracycline [136,137]. In field-growing coconuts, palms can be treated with trunk-injections of oxytetracycline at doses ranging from 50 to 20 g L^−1^. This is done when LY symptoms begin to appear. However, it is known that low soil pH and poor antibiotic translocation can lower the efficacy of these antibiotic treatments [97]. However, widespread application of antibiotics is currently not a feasible option for large-scale control of the LY phytoplasma due to their cost and their potential harm to the farming community. In Australia, the use of antibiotics on food-producing plants is prohibited for fear of the evolution of antimicrobial resistance [138]. In in vitro systems, an antibiotic preculture may serve as an initial treatment to reduce phytoplasma contamination. However, the effectiveness of chemotherapy is currently under-investigation in the coconut. So far, no antiviral substances have been reported to be effective in eliminating coconut viruses, while the reappearance of symptoms within various horticultural crops once chemotherapy/antibiotic treatments are stopped shows that more effective methods still need to be developed [134]. 

### 5.2. Thermotherapy and Cryotherapy

Several in vitro phytoplasma and virus elimination methods have been developed, that typically use a combination of steps in their application. The most effective methods incorporate either a thermotherapy or a cryotherapy step (e.g., for grapevine, *Vitis vinifera* L. [139,140]) with a previously applied meristem culture step. Thermotherapy, the oldest and most well-known virus elimination method [141], uses heat (generated through air or water) to treat infected tissues. This is often applied before the meristems are excised to eliminate endogenous viruses (e.g., raspberry, *Rubus idaeus* L. [142]), phytoplasma, or other bacterial diseases (in various horticultural crops [143]). On the other hand, cryotherapy (the use of ice crystal formation caused by ultra-low temperatures to eliminate infected cells) has been proven to eliminate pathogens in some plant species in tissue culture materials, such as banana (*Musa* spp.; [144], sweet potato (*Ipomoea batatas* L. [145]) and grapevine (*Vitis* sp. [146]). Although the application of thermotherapy or cryotherapy has not been applied to coconut meristems yet [147], a cryopreservation technique for coconut meristems has been developed [70,148]. The technique of a meristem culture coupled with thermotherapy or cryotherapy could eliminate pathogens in coconut meristems during the germplasm exchange process. It will be essential to determine the efficacy of any elimination technique prior to its implementation, especially when using cryotherapy, as facilities to do this are currently not available in many countries.

### 5.3. Limitations of Elimination Methods

Antibiotic treatments for diseases like LY in coconuts are impractical as they cannot be used for large-scale application and do not have long-term effectiveness. For thermotherapy, it necessary to combine it with a more technically difficult tissue culture method like a meristem culture, while cryotherapy approaches are new and therefore require more validation to ensure global safety and efficacy. It is also important to point out that certain components used in a cryotherapy system (e.g., cryoprotectants) may not deactivate viruses but instead preserve them [149,150], so this method can lead to unwanted consequences if misused (Table 2 and Table 3). 

## 6. Research Gaps and Research Priorities towards Safe Exchange of Coconut Germplasm

Transboundary disease transmissions have been a problem for generations and now are thought to be exacerbated by climate change [151]. The effects of a lethal coconut pathogen introduction to a new country would be devastating with long-lasting effects on multiple socioeconomic levels. Informed through various case studies, undertaken on several species, and from some limited previous research on coconut disease detection and elimination, several key research gaps are identified that need to be filled to enable safe international exchange of coconut germplasm.

### 6.1. Lack of Knowledge on Vertical Transmission of Lethal Coconut Pathogens, and Their Detection in Various Tissues

Certain seed-borne pathogens may survive through the stages of zygotic embryo development and may remain functional in the resulting field-growing plant. However, limited knowledge is available concerning pathogenic agent transfer through the presently used coconut germplasm exchange system. This is extremely important and needs to be determined for all the major lethal diseases of the coconut before safe exchange of coconut germplasm can be assured.

**Figure 2:**
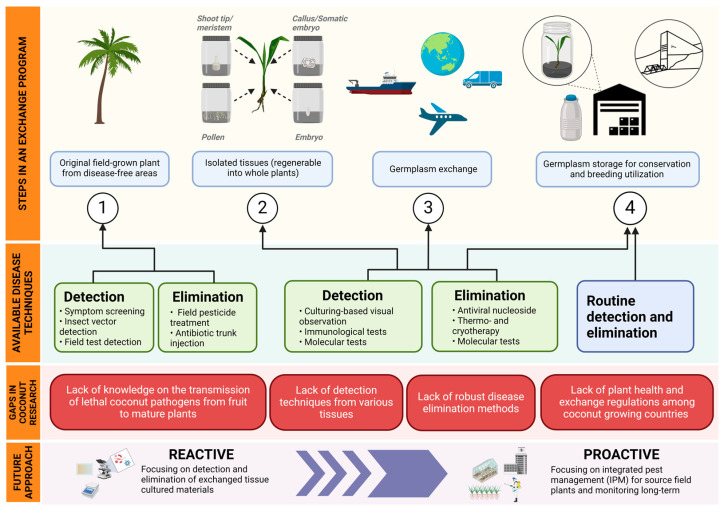
A conceptual summary of the steps that should be considered for an ideal coconut germplasm exchange program. The diagram shows the pathogen detection and elimination methods that should be used, the times when these and other methods should be applied in the program, the current gaps in coconut research along the exchange timeline, and a recommended reactive and proactive approach for disease management (Figure created with BioRender.com).

Although several methods exist to detect the presence of pathogens in various tissues, they are not presently being used in coconut germplasm exchange. It is possible that fruit may be inadvertently harvested from diseased palms, and therefore certain pathogens may be present in those embryos being exported. Thus, it is recommended that all palms should be field-tested for the major coconut diseases before fruit is harvested, and the materials to be exchanged should then be subjected to further laboratory testing for pathogens by using a combination of techniques before being exported. If this is not possible, certainly this must be undertaken upon importation and while the tissues are still under quarantine. This would require the development of reliable, sensitive, accurate, and rapid diagnostic methods for all lethal coconut diseases (Figure 2). The infrastructure capacity and skilled personnel will be key for this procedure to progress in developing countries. 

### 6.2. Lack of Robust Disease Elimination Methods

The customary practice when disease is detected in an imported coconut germplasm shipment is to discard that shipment and source a new one. However, a more convenient and cost-effective approach would be to eliminate the disease-causing agents. Even though the presence of certain pathogens has been reported in coconut embryos, no studies have been undertaken to eliminate them from those tissues. Given the well-documented successes in pathogen eradication across various species using a meristem culture in conjunction with thermotherapy or cryotherapy, this presents a promising area for further research in pathogen elimination in the coconut. However, it is important to acknowledge that only one report presently exists regarding the successful conversion of isolated coconut meristems into field-grown plants [70]. Therefore, an additional effort is now required to develop pathogen elimination methods for the coconut by implementing some approaches that have been developed for other species (Figure 2).

### 6.3. Lack of Plant Health and Exchange Regulations among Coconut-Growing Countries

In the past, the international transfer of coconut germplasm has been interrupted on several occasions. An infestation of CCCVd in the central Philippines [152], WCLW in Sri Lanka [153], and BCS in PNG [22] prevented any living parts of the coconut palm to be moved out of the affected area. These recurring interrupted actions in germplasm transfer could have been prevented with a functional disease-control program in place, with geographically relevant guidelines for germplasm testing and transfer.

The present guidelines for coconut germplasm exchange were prepared at a time when there was a poor understanding of coconut pathogen biology [154]. Although global regulations regarding plant health have been in existence for many years [155], no proper regulations have been developed for the international exchange of coconut germplasm (Table 4). Hence, the methods used are highly variable among coconut-growing countries. Currently, the biosecurity requirements surrounding the international exchange of coconut germplasm are based on the technical guidelines published by FAO in partnership with IBPGR/IPGRI, with further advice in the guidelines developed by the Coconut Genetic Resources Network (COGENT) and Alliance of Bioversity International and the International Center for Tropical Agriculture (CIAT) (Table 5). The scientific insights made from these presently identified research gaps should now be integrated to refine and update these guidelines. This will enable safe coconut germplasm exchange between countries.

## 7. Conclusions

At present, the use of superior coconut germplasm to replant areas after the removal of diseased and senile coconut palms is important as it promises significant economic gains for smallholder farmers as well as uplifting the various industries dependent upon coconut cultivation. To achieve this goal, it is necessary for coconut-growing countries to explore, conserve, and share highly productive coconut germplasm to enhance breeding efforts. It had been considered that the in vitro transfer of zygotic embryos, taken from disease-free areas, was the most promising method for the exchange of pathogen-free germplasm. However, in other species, it is now known that phytoplasma, viroid, and virus diseases can be transmitted in fruit and, in some instances, seed tissues. This has led to a concern that this may also occur in coconut germplasm exchange when zygotic embryos are used, especially because LY phytoplasma has been detected in coconut embryo tissues when isolated from infected palms. Hence, it is imperative to test the materials for the presence of pathogens at least once during the exchange process. 

Although there is no evidence of disease introduction following coconut germplasm exchange using the in vitro embryo culture system, recent studies suggest that this could occur in the future. Hence, it is necessary to develop appropriate pathogen testing methods, several of which are now available. As every method has its own advantages and disadvantages to accurately detect pathogens, future research should focus on the development of a combinational testing approach (e.g., using a microscopical with a molecular method or a qPCR with an NGS approach) and ensure that they are functional on a wide range of coconut tissues. In addition, it is important that certain identified research gaps are filled to ensure the continued safety of germplasm exchange. The research gaps identified suggest that a better understanding is required on how lethal pathogens can be transmitted through the in vitro exchange of embryos, how reliable, sensitive, and robust are the methods available for disease detection, and how it would be possible to eliminate pathogens from tissues if they are present. Once these research areas are studied, improved guidelines and regulations should be developed to facilitate the safe exchange of coconut germplasm between countries. These efforts need to be undertaken urgently to meet the ever-increasing demand for coconuts around the world.

## Figures and Tables

**Table 2:** Suggested methods for coconut pathogen detection.

Method	Type	Targeted Coconut Pathogens	Source
Field transmission	Artificial transmission test systems	Phytoplasma	[43,81]
In vitro transmission	Artificial transmission test systems	Phytoplasma	[20,83]
Transmission electron microscope (TEM)	Visual	Viroids, virus, phytoplasma	[86]
Fluorescent stain 4′,6-diamidino-2-phenylindole (DAPI).	Visual	Phytoplasma	[88]
ELISA assay	Immunological	Phytoplasma	[93,94,95]
Antibiotic response	Immunological	Phytoplasma	[97]
Hybridization assay	Molecular	Viroids, phytoplasma	[100]
Polymerase chain reaction (PCR)-based methods	Molecular	Virus, viroids, phytoplasma	[15,24,83,99,102,103,104,105,111,112,113,114,115,116]
Isothermal amplification techniques (IAT)	Molecular	Virus, viroids, phytoplasma	[126,127,128,129]
Next-generation sequencing (NGS)	Molecular	Not yet studied in coconut	[106,130]

**Table 3:** Suggested methods for coconut pathogen elimination.

Methods	Targeted Pathogens	Targeted Coconut Pathogens	Source
Chemotherapy	Phytoplasma	Phytoplasma	[136,137]
Thermotherapy	Virus	Not yet studied in coconut	[142,143]
Cryotherapy	Virus	Not yet studied in coconut	[144,145,146,147]

**Table 4:** The existing guidelines and safety protocols for coconut germplasm exchange.

Name of Guideline	Source	Year Published
FAO/IBPGR Technical Guidelines for the Safe Movement of Coconut Germplasm	[154]	1993
Germplasm Health Management for COGENT’s Multi-Site International Coconut Genebank	[156]	2004
COGENT Global Conservation Strategy for Cocos Nucifera: A Framework for Promoting the Effective Conservation and Use of Coconut Genetic Resources Developed in Consultation with COGENT Members and Partners	[157]	2008
Technical guidelines for the safe movement and duplication of coconut (*Cocos nucifera* L.) germplasm using embryo culture transfer protocols	[158]	2012
A Global Strategy for the Conservation and Use of Coconut Genetic Resources, 2018–2028: Summary Brochure	[159]	2018
Coconut risk management and mitigation manual for the Pacific Region	[160]	2021

**Table 5:** Progress in the development of guidelines for coconut germplasm exchange and the advancement in the understanding of coconut phytoplasma, virus, and viroid diseases.

Year	Coconut Germplasm Guidelines	Advances/Gaps in the Understanding of Phytoplasma, Virus and Viroid Diseases
Before 2003	Coconut fruit (dehusked and surface-sterilized), some zygotic embryos and pollen were the main internationally exchanged materials. The guidelines stated that pollen had to be tested for the presence of phytoplasma, and de-husked fruit and in vitro cultured embryos needed to be tested for viruses, viroids, or insect pests [154]. The embryos should first be cultured in vitro in the country of origin rather than the receiving country. If the country of origin or the recipient lacks the necessary tissue culture facilities, embryos should be sent to a different, third country for their culture [154].	Phytoplasma diseases were still largely unexplored. Coconut husk, shell, and endosperm tissues were all shown to contain the Coconut cadang-cadang viroid [45]. DNA of the Coconut foliar decay virus was detected on the coconut fruit husk and in zygotic embryos but was not shown to be transferred through zygotic embryo cultures [161].
2003–2005	Germplasm should be distributed in an in vitro form. No strict management guidelines were developed for the donor or recipient countries for zygotic embryos and other in vitro transferred materials. Tissues should not be sourced from disease-stricken areas, and pathogen testing should be undertaken. Pest risk analysis approaches were introduced to assist in pest control [156]. Food and Agriculture Organization/The International Board for Plant Genetic Resources (FAO/IBPGR) released technical guidelines for the safe movement of coconut germplasm, which recommended the transfer of isolated zygotic embryos instead of whole or de-husked fruit [137,156].	Lethal yellowing phytoplasma DNA was detected in embryos extracted from diseased fruit by in situ PCR [38].
2005–2012	The International Coconut Genetic Resources Network (COGENT) adopted the FAO/IBPGR guidelines and incorporated them into its global strategy for 2005–2015 and designated the embryo culture as a key technique to be further refined for the international exchange of coconut germplasm [157]. Pollen transfer was identified as having fewer quarantine complications than other forms of tissue transfer [157]. A general recommendation was made for the transfer of in vitro coconut materials, including a full procedure for the transfer of zygotic embryos. The recommendation was that in vitro germination should be undertaken in the donor country and the ex vitro growth take place in the recipient country [153,158]	Studies show that some embryos taken from phytoplasma-diseased coconut palms do not contain phytoplasma DNA [15,112]. Pollen is reported to transmit several lethal palm virus and viroid diseases [162].
2012–2020	Guidelines were developed that required International genebanks to duplicate their collections in other geographical locations due to the threat from phytoplasma diseases [159].	Phytoplasma DNA was detected in the plumules of in vitro germinated coconut embryos coming from infected fruit [20]. Bogia coconut syndrome and Banana wilt-associated phytoplasma DNA were shown to be introduced to coconut palms when caged with phytoplasma-carrying insects [22].
2021-present	Pollen transfer was still allowed; however, suspicion was raised about embryo culture; current embryo exchange activities became restricted. Further transmission confirmation research is now considered necessary for zygotic embryo transfer [160].	An in vitro assay was developed to confirm the transmission of Lethal yellowing phytoplasma by planthoppers (*Halaxius crudus*) onto in vitro growing coconut seedlings, 16SrIV-group phytoplasmas were detected by nested PCR [83].

## Data Availability

All data generated or analyzed during this study are included in this published article.
